# Aromatic Polyketides from a Symbiotic Strain *Aspergillus fumigatus* D and Characterization of Their Biosynthetic Gene *D8.t287*

**DOI:** 10.3390/md18060324

**Published:** 2020-06-20

**Authors:** Yi Hua, Rui Pan, Xuelian Bai, Bin Wei, Jianwei Chen, Hong Wang, Huawei Zhang

**Affiliations:** 1 School of Pharmaceutical Sciences, Zhejiang University of Technology, Hangzhou 310014, China; huayi@zjut.edu.cn (Y.H.); ruipan1109@163.com (R.P.); binwei@zjut.edu.cn (B.W.); cjw983617@zjut.edu.cn (J.C.); 2 College of Life and Environmental Sciences, Hangzhou Normal University, Hangzhou 310036, China; baixl2012@163.com

**Keywords:** *Aspergillus fumigatus*, symbiotic microbe, aromatic polyketide, genome sequencing, biosynthesis

## Abstract

The chemical investigation of one symbiotic strain, *Aspergillus fumigatus* D, from the coastal plant *Edgeworthia chrysantha* Lindl led to the isolation of eight compounds (**1**–**8**), which were respectively identified as rubrofusarin B (**1**), alternariol 9-*O*-methyl ether (**2**), fonsecinone D (**3**), asperpyrone A (**4**), asperpyrone D (**5**), fonsecinone B (**6**), fonsecinone A (**7**), and aurasperone A (**8**) by a combination of spectroscopic methods (1D NMR and ESI-MS) as well as by comparison with the literature data. An antimicrobial assay showed that these aromatic polyketides exhibited no remarkable inhibitory effect on *Escherichia coli*, *Staphyloccocus aureus* and *Candida albicans*. The genomic feature of strain D was analyzed, as well as its biosynthetic gene clusters, using antibiotics and Secondary Metabolite Analysis Shell 5.1.2 (antiSMASH). Plausible biosynthetic pathways for dimeric naphtho-*γ*-pyrones **3**–**8** were first proposed in this work. A non-reducing polyketide synthase (PKS) gene *D8.t287* responsible for the biosynthesis of these aromatic polyketides **1**–**8** was identified and characterized by target gene knockout experiment and UPLC-MS analysis.

## 1. Introduction

**Figure 1:**
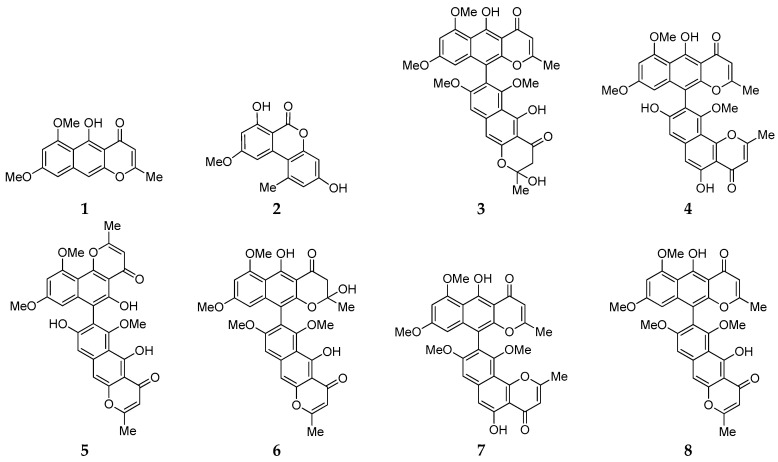
Chemical structures of compounds **1**–**8** from strain D.

Symbiotic microorganisms are generally acknowledged as a significant source of structurally novel and biologically diverse chemicals, including alkaloids, terpenoids, polyketides, depsipeptides and perylenequinonoid derivatives [1]. The genus *Aspergillus* possesses strong potential to biosynthesize cryptic secondary metabolites (SMs) with prominent biological properties, ranging from antioxidant, to anti-bacteria, to anti-cancer drugs [2,3,4]. Strain D was isolated from coastal plant *Edgeworthia chrysantha* Lindl. grown on the coast of Hangzhou Bay (China) and identified as *A. fumigatus* based on its morphological characteristics and analyses of the 18*S* rDNA gene sequence (GenBank accession No. KR019681) [5]. Bioassay-guided fractionation of the ethyl acetate extract of strain D, cultured in salted and unsalted Czapek media, previously led to the isolation of seven antimicrobial agents, including bisdethiobis (methylthio) gliotoxin, gliotoxin, pseurotin A, and spirotryprostatins A and G, ACTG-toxin F, and 8-chloro-3,6a,7,9,10-pentahydroxy-9,8,7,6a-tetrahydroperylen-4(6a*H*)-one [6,7,8]. In our continuous search for more bioactive SMs from strain D using the “one strain many compounds” (OSMAC) strategy [9], chemical investigation was further carried out in this work. Herein, we reported the discovery of eight known aromatic polyketides (**1**–**8**) (Figure 1) from strain D when grown on a rice medium, and the characterization of a vital biosynthetic gene encoding polyketide synthase (PKS) by target gene knockout and UPLC-MS analysis.

## 2. Results

### 2.1. Isolation, Identification and Antimicrobial Assay of Aromatic Polyketides from Strain D

Chemical study of the ethyl acetate extract of fermented rice of strain D resulted in the isolation of eight SMs (**1**–**8**) using HPLC C18 columns. By a combination of spectroscopic analysis (^1^H and ^13^C NMR and ESI-MS) and comparison with the literature data (Tables S3–S10), these compounds were unambiguously identified as rubrofusarin B (**1**) [10], alternariol 9-*O*-methyl ether (**2**) [11], fonsecinone D (**3**) [12,13], asperpyrone A (**4**) [14], asperpyrone D (**5**) [15], fonsecinone B (**6**) [12], fonsecinone A (**7**) [16], aurasperone A (**8**) [17], respectively. Biological tests indicated that no compound exhibited potent antimicrobial activity against human pathogenic microbes *Escherichia coli*, *Staphyloccocus aureus* and *Candida albicans* with minimum inhibitory concentration (MIC) values of ≥100 µM.

### 2.2. Genome Features of Strain D

Genome sequencing of strain D was performed on a PacBio RSII platform according to the reported procedure [18,19]. After gene sequence assembly, a single linear chromosome with a size of 33.40 Mb and an average G + C content of 51.4% was obtained (Table 1). By comparison with ten other genomes of *A. fumigatus* strains deposited at National Center for Biotechnology Information (NCBI) database, the genome of strain D has a larger size and a higher G + C content. However, it possesses the smallest number of conserved hypothetical proteins and tRNA genes. In addition, the genome of strain D contains a similar number (9789) of protein-encoding genes.

### 2.3. Biosynthesis Gene Cluster (BGC) Analysis of Secondary Metabolite in Strain D

**Figure 2:**
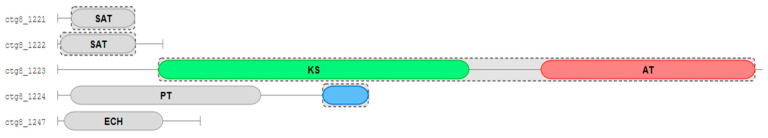
Domain annotation of the non-reducing PKS gene *ctg8_1223* in strain D by antiSMASH. (AT: acyltransferase; ECH: enoyl-CoA hydratase; KS: ketoacylsynthase; PT: phosphotransferase; SAT: serine acetyltransferase).

The complete genome sequence of strain D was submitted to antiSMASH (antibiotics and Secondary Metabolite Analysis Shell 5.1.2) [20]. A total of 22 putative biosynthesis gene clusters (BGCs) were found, including seven PKS gene clusters, 10 NRPS (non-ribosome peptide synthetase) and its related gene clusters, four terpene and one fungal-*RiPP* (ribosomally synthesized and post-translationally modified peptide) synthetase gene clusters (Table 2). It suggested that strain D had the potential capability to biosynthesize polyketides, alkaloids or nitrogenous-containing compounds, and terpene derivatives. However, only 10 BGCs were identified as the most similar to the known clusters in antiSMASH analysis. It was noteworthy that the gene cluster scaffold8.2 has the same sequence as that of BGC biosynthesizing 1,3,6,8-tetrahydroxynaphthalene (T4HN) in *Glarea lozoyensis*, which was shown to encode iterative type I PKS [21]. Therefore, the gene *ctg8_1223* in the gene cluster scaffold8.2 was predicted to encode the non-reducing PKS containing two domains, KS (ketoacylsynthase) and AT (acyltransferase), in strain D (Figure 2). These aromatic polyketides **1**–**8** might be biosynthetically regulated by the gene *ctg8_1223* [22].

### 2.4. Biosynthesis Analysis of Compounds ***1**–**8*** in Strain D

Diamond (v0.9.10.111) was used to identify sequence similarities of all genes in the genome of strain D with previously known related genes. Basic Local Alignment Search Tool (BLAST) results indicated that nine genes were characterized as core PKS biosynthetic genes (Table 3). The gene *D8.t287* has the most similar position (938,512–942,754) to that of the predicted gene *ctg8_1223* (938,158–941,487) inside scaffold8.2. Furthermore, *D8.t287* has high sequence similarity (85.3%) with the gene *alb1*, which had been shown to encode naphthopyrone synthase using heterologous expression in *A. oryzae* [23]. Therefore, the gene *D8.t287* plays a vital role in encoding KS and AT followed by the biosynthesis of these aromatic polyketides **1**–**8** in strain D.

**Figure 3:**
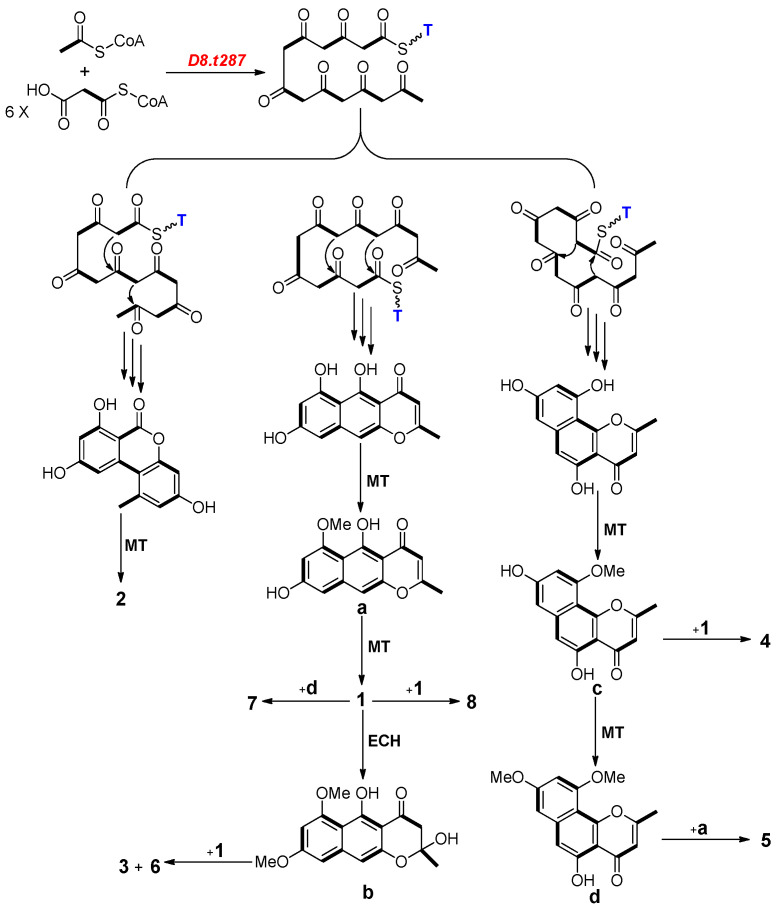
Plausible biosynthetic pathways for compounds **1**–**8** in strain D. (MT: methyltransferase; ECH: enoyl-CoA hydratase; T: thiolation).

As shown in Figure 3, possible biosynthetic pathways for aromatic polyketides **3**–**8** were first proposed in this study. One acetyl-CoA and six malonyl-CoA were used as substrata for the biosynthesis of compounds **1**, **2** and four intermediates **a**–**d** by successive catalytic reactions in a non-reducing PKS system [24,25]. Two of these naphtho-γ-pyrones **1** and **a**–**d** further dimerized at various carbon positions (C-6, C-7, C-9 or C-10) and resulted in the formation of compounds **3**–**8**. Therefore, *D8.t287* was deduced to be the key PKS gene responsible for the biosynthesis of the initial precursor heptaketone of all aromatic polyketides.

### 2.5. Functional Verification of the PKS Biosynthetic Gene Scaffold8.t287

**Figure 4:**
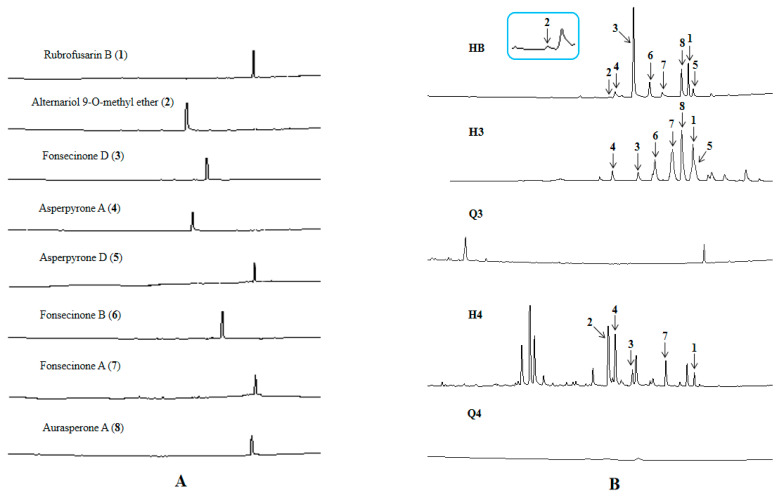
Comparison of UPLC profiles of crude extracts of strains D and ∆HY44. (**A**: compounds **1**–**8**; **B**: HB, mixture of compounds **1**–**8**; H3 and H4: fractions of the crude extract I of wild strain D; Q3 and Q4: fractions of crude extracts II of mutant strain ∆HY44).

The target PKS biosynthetic gene *D8.t287* was successfully knocked out and a mutant strain ∆HY44 was obtained by transformation experiments (Figures S2–S5). Fermentation of the strain ∆HY44 was carried out and six fractions (Q1–Q6) of crude extract were prepared using the same procedure as with those of strain D as described in Section 3.3. UPLC-MS analysis indicated that all aromatic polyketides were detected in fractions H3 and H4 of the crude extract of strain D except compound **2** owing to its trace amount (Figure 4). However, none of these aromatic polyketides were found in fractions Q3 and Q4 of strain ∆HY44. Therefore, the gene *D8.t287* in strain D was identified as the key PKS gene encoding KS and AT, which were further responsible for biosynthesis of these aromatic polyketides **1**–**8**.

## 3. Materials and Methods

### 3.1. General Experimental Procedures

NMR spectra were analyzed on a Bruker 600 MHz Avance III HD spectrometer (Bruker, Fällande, Switzerland) equipped with a triple resonance probe at 298 K, using TMS (trimethylsilane) as an internal standard. ESI-MS data were taken on an Agilent 6210 LC-MS spectrometer (Agilent Technologies, Santa Clara, CA, USA). Purification of all compounds was performed on Waters D600 apparatus (Waters, San Diego, CA, USA), Agilent 1200 apparatus (Agilent, USA), equipped with a preparative column (Phenomenex Gemini-NX, 50 × 21.2 mm), a semi-preparative column (Agilent Eclipse XDB-C18, 9.4 × 250 mm, 5 µm, USA), and an analytical column (Synergi Hydro-RP, 250 mm × 4.6 mm, 4 µm, Phenomenex, Torrance, CA, USA). The acetonitrile (Merck, Darmstadt, Germany), methanol (Sigma-Aldrich Co., St. Louis, MO, USA) and H_2_O that were used in HPLC system were of chromatographic grade, and all other chemicals were analytical.

### 3.2. Strain, Medium and Cultural Conditions

Strain D was isolated as an endophytic fungus from the healthy leaves of *E. chrysantha* Lindl., which naturally grew on the bank about 10 m from intertidal zone of Hangzhou Bay (China) in 2015. It was identified as *A. fumigatus* using its morphological characteristics and analysis of 18*S* rDNA gene sequence (GenBank accession No. KR019681) as described before [6]. Previous chemical investigation indicates that strain D could grow well in salted Czapek medium with 3% or 10% NaCl, and produce various secondary metabolites, which had been reported [7,8]. In antimicrobial tests, three human pathogenic strains, *E. coli* AB 94012, *S. aureus* AB 2010021 and *C. albicans* AY 204006, were purchased from the China Center for Type Culture Collection (CCTCC). In the gene knockout experiment, *Agrobacterium tumefaciens* (*Agl*) and *E. coli* DH5α were routinely grown on an Luria-Bertani medium. The plasmid pAF-G418 was kindly provided by Dr Jintao Cheng from Zhejiang University, China.

### 3.3. Fermentation, Extraction and Isolation

Firstly, strain D was cultured on Potato Dextrose Agar (PDA) at 28 °C for 7 days. A balanced amount of fungal colony was transferred to the culture broth in a 500 mL Erlenmeyer flask, which contained 250 mL Potato Dextrose Broth (PDB) consisting of potato 200 g·L^−1^, glucose 20 g·L^−1^. This was followed by shaking at 200 rpm at 28 °C for 3 days that prepared it as seed broth; then the seed broth was transferred to solid medium in 500 mL Erlenmeyer flask, which contained 80 g of rice and 160 mL of water, cultured at 28 °C for 40 days. At the end of fermentation, all medium was extracted twice with double the volume of ethyl acetate (Merck); the broth was collected and filtered through gauze, which afforded the filtrate (approximate 68 L). The upper solvent was evaporated at 25 °C in a vacuum to yield extract I (about 82.22 g), followed by its separation on a preparative HPLC column to afford six fractions (H1–H6) under a gradient condition of CH_3_CN in H_2_O, with a flow rate of 10.0 mL·min^−1^ and 254 nm detection. Fraction H3 was then separated under a gradient condition of CH_3_CN in H_2_O, with a flow rate of 3.5 mL·min^−1^, and 254 nm detection to get eight fractions (H3A–H3H) (Figure S1). Bioactive fractions H3B-H3E were further subjected to HPLC with an analytical HPLC column (1.0 mL/min) to give compounds **1** (2.0 mg, 0.0024%), **2** (1.0 mg, 0.0012%), **3** (1.3 mg, 0.0016%) and **4** (1.0 mg, 0.0012%) under an isocratic condition of 65% CH_3_OH, with a flow rate of 1.0 mL/min at 254 nm. Compounds **5** (2.0 mg, 0.0024%), **6** (5.2 mg, 0.0063%), **7** (1.8 mg, 0.0022%) and **8** (3.0 mg, 0.0036%) were purified from bioactive fractions H3F-H3H using a semi-preparative HPLC column, under an isocratic condition of 70% CH_3_OH.

Rubrofusarin B (**1**): Yellowish amorphous powder; C_16_H_14_O_5_; ^1^H NMR (600 MHz, CDCl_3_): 2.38 (3H, *s*), 3.94 (3H, *s*), 4.02 (3H, *s*), 6.01 (1H, *s*), 6.41 (1H, *d*, *J* = 2.35), 6.60 (1H, *d*, *J* = 2.35), 6.98 (1H, s), 14.98 (1H, *s*, 5-OH). ^13^C NMR (600 MHz, CDCl_3_): 23.81, 64.38, 66.30, 97.37, 97.93, 101.15, 104.50, 107.45, 141.40, 149.18, 160.76, 161.61, 162.76, 167.80, 183.90. ESI-MS: 287 [M + H]^+^.

Alternariol 9-*O*-methyl ether (**2**): Yellowish amorphous powder; C_15_H_12_O_5_; ^1^H NMR (600 MHz, DMSO-*d*_6_): 2.73 (3H, *s*), 3.91 (3H, *s*), 6.62 (1H, *d*, *J* = 1.90), 6.65 (1H, *d*, *J* = 2.30), 6.73 (1H, *d*, *J* = 2.30), 7.22 (1H, *d*, *J* = 1.90). ^13^C NMR (600 MHz, CDCl_3_): 25.49, 55.92, 98.94, 102.01, 103.86, 109.53, 118.13, 136.18, 138.28, 159.32, 165.16, 166.64. ESI-MS: 271 [M − H]^−^.

Fonsecinone D (**3**): Yellowish amorphous powder; C_32_H_28_O_11_; ^1^H NMR (600 MHz, CDCl_3_): 1.83 (3H, *s*), 2.13 (3H, *s*), 3.02/3.07 (2H, *s*), 3.44 (3H, *s*), 3.65 (3H, *s*), 3.78 (3H, *s*), 4.04 (3H, *s*), 6.00 (1H, *d*, *J* = 4.08), 6.23 (1H, *d*, *J* = 2.49), 6.43 (1H, *d*, *J* = 2.09), 6.73 (1H, *d*, *J* = 1.41), 6.86 (1H, *d*, *J* = 1.41), 14.17 (1H, *s*), 15.24 (1H, s). ESI-MS: 589 [M + H]^+^.

Asperpyrone A (**4**): Yellowish amorphous powder; C_31_H_24_O_10_; ^1^H NMR (600 MHz, CDCl_3_): 2.10 (3H, *s*), 2.48 (3H, *s*), 3.39 (3H, *s*), 3.58 (3H, *s*), 3.93 (3H, *s*), 6.14 (1H, *d*, *J* = 2.29), 6.20 (1H, *s*), 6.50 (1H, *s*), 6.55 (1H, *d*, *J* = 2.28), 6.95 (1H, *s*), 7.06 (1H, *s*), 12.90 (1H, *s*), 15.10 (1H, *s*). ESI-MS: 557 [M + H]^+^.

Asperpyrone D (**5**): Yellowish amorphous powder; C_31_H_24_O_10_; ^1^H NMR (600 MHz, CDCl_3_): 2.41 (3H, *s*), 2.58 (3H, *s*), 3.65 (3H, *s*), 3.66 (3H, *s*), 4.03 (3H, *s*), 6.04 (1H, *s*), 6.32 (1H, *d*, *J* = 2.21), 6.37 (1H, *s*), 6.49 (1H, *d*, *J* = 2.21), 7.11 (1H, *s*), 7.18 (1H, *s*), 13.47 (1H, *s*), 14.87 (1H, *s*). ESI-MS: 555 [M − H]^−^.

Fonsecinone B (**6**): Yellowish amorphous powder; C_32_H_28_O_11_; ^1^H NMR (600 MHz, CDCl_3_): 1.50 (3H, *s*),2.43 (3H, *s*), 3.45 (3H, *s*), 3.66 (3H, *s*), 3.84 (3H, *s*), 4.02 (3H, *s*), 6.07 (1H, *s*), 6.15 (1H, *d*, *J* = 2.21), 6.38 (1H, *d*, *J* = 2.21), 7.00 (1H, *s*), 7.16 (1H, *s*), 14.55 (1H, *s*), 14.81 (1H, *s*). ESI-MS: 587 [M − H]−.

Fonsecinone A (**7**): Yellowish amorphous powder; C_32_H_26_O_10_; ^1^H NMR (600 MHz, CDCl_3_): 2.12 (3H, *s*), 2.48 (3H, *s*), 3.43 (3H, *s*), 3.61 (3H, *s*), 3.78 (3H, *s*), 4.03 (3H, *s*), 6.00 (1H, *s*), 6.19 (1H, *d*, *J* = 2.20), 6.33 (1H, *s*), 6.43 (1H, *d*, *J* = 2.20), 6.97 (1H, *s*), 12.83 (1H, *s*), 15.24 (1H, *s*). ^13^C NMR (600 MHz, CDCl_3_): 20.72, 20.85, 55.33, 56.14, 56.38, 61.37, 96.43, 97.14, 101.71, 104.40, 105.16, 106.18, 107.52, 108.14, 108.75, 109.54, 110.80, 117.29, 140.78, 140.93, 150.98, 155.23, 156.82, 157.05, 160.16, 161.25, 161.72, 162.95, 167.00, 167.62, 183.08, 184.70. ESI-MS: 571 [M + H]^+^.

Aurasperone A (**8**): Yellowish amorphous powder; C_32_H_26_O_10_; ^1^H NMR (600 MHz, CDCl_3_): 2.12 (3H, *s*), 2.41 (3H, *s*), 5.98 (1H, *s*), 6.05 (1H, *s*), 14.83 (1H, *s*), 3.46 (3H, *s*), 3.62 (3H, *s*), 3.78 (3H, *s*), 4.02 (3H, *s*), 6.97 (1H, *s*), 7.15 (1H, *s*), 2.12 (3H, *s*), 5.98 (3H, *s*), 15.24 (1H, *s*), 4.02 (3H, *s*), 6.41 (1H, *d*, *J* = 2.27), 3.62 (3H, *s*), 6.20 (1H, *d*, *J* = 2.27). ESI-MS: 571 [M + H]^+^.

### 3.4. Antimicrobial Test

Antimicrobial activity was assessed by the microbroth dilution method in 96-well microtitreplates [26]. Antibiotics ampicillin and amphotericin B (Sigma-Aldrich, Buchs, Switzerland), were used as positive controls, and an equivalent amount of DMSO was used as a negative control. The tested bacteria were cultured in the LB medium for 24 h at 37 °C at 150 rpm, and the tested fungus was incubated in the Sabouraud medium for 48 h at 28 °C at the same rotatory speed. A bacteria or fungi suspension of 1 × 10^6^ cfu/mL was applied to evaluate the antimicrobial activities of pre-HPLC derived fractions and all purified compounds. Testing solution at the initial concentration of 100 µM (100 µL) was added to 96-well microplate. Two-fold serial dilutions were made in the 96-well round-bottom sterile plates and then 100 µL of the microbial suspension was added. After incubation, MIC was taken as the lowest concentration of each test compound in the wells of the 96-well plates, in which the lowest microbial growth could be measured at 600 nm. All tests were carried out in triplicate.

### 3.5. Genome Sequencing and Analysis

Strain D was cultured in PDB, which was filtered in a vacuum until dry and the cells were ground under liquid nitrogen. Genomic DNA for both sequencing and PCR analysis was prepared using the TruSeqTM DNA Sample Prep Kit. Genome sequencing and assembly was performed at Shanghai Personal Biotechnology Co., Ltd. (China) using PacBio RSII platform [27].

### 3.6. Gene D8.t287 Knockout Experiment

#### 3.6.1. Screening Resistance Markers

The Gene *D8.t287* knockout experiment consisted of a resistance marker screen, the construction of a knockout vector, and the transformation and verification of the mutant strain. The neo gene and hyg gene respectively conferring genetin (G418) and hygromycin B (Hyg) resistance were used as the selector markers for transformed fungus [28]. Spores of the strain D were prepared by inoculation of the fungus onto PDA plates, followed by cultivation on PDA supplemented with G418 (at final concentrations of 0, 25, 50, 100, 150 and 200 μg·mL^−1^) and Hyg (at final concentrations of 0, 25, 50, 100, 150 and 200 μg·mL^−1^). The results indicated that strain D was sensitive to Hyg and G418, and its growth was halted on PDA containing at least 25 μg·mL^−1^ of Hyg or 150 μg·mL^−1^ of G18 (Figure S2). Finally, G418 was selected as the resistance marker in subsequent experiments.

#### 3.6.2. Construction of a Knockout Vector

The genome of strain D was obtained by the CTAB (cetyl trimethyl ammonium bromide) method [29]. Two pairs of primers were designed and the fragments LB and RB on both sides of the target gene *D8.t287* were gained using PCR reactions (Table S1). The sequences of all primers are showed in Table S2. The amplification reaction was carried out at 95 °C for 5 min, 94 °C for 30 s, and 25 cycles of 72 °C for 10 min, followed by 4 °C for 8 min. The vector pAF-G418 was digested with the restriction enzyme EcoRI, followed by its combination with the fragment LB by seamless cloning kit. Then, the combined vector pAF-G418-LB was transferred into *E. coli* DH5α, and positive transformants were cultured, screened and verified by PCR (Figure S3). Then, vector pAF-G418-LB was extracted and combined with fragment RB by digestion and the use of a seamless cloning kit. Finally, four of the ten selected transformants containing pAF-G418-HY44-left+right (pHY44-L+R) grew well on the G418 resistant plate and were determined to be positive by PCR verification (Figure S4).

#### 3.6.3. Transformation and Verification of Mutant Strain

Recombinant vectors were extracted from positive transformants of *E. coli* DH5α and incubated with *Agrobacterium tumefaciens* in LB broth containing kanamycin (100 μg·mL^−1^) for 24 h at 200 rpm and 28 °C. Then the cell suspension was centrifuged, and the harvested cells were resuspended in an Induction Minimal Medium (IMM) containing 200 μM acetosyringone (AS) for 6 h to an OD_600_ value of 0.60. The IMM contained glucose 1 gL^−1^, KH_2_PO_4_ 2.05 gL^−1^, NH_4_NO_3_ 0.5 gL^−1^, CaCl_2_ 0.01 gL^−1^, MgSO_4_·7H_2_O 0.6 gL^−1^, NaCl 0.3 gL^−1^, FeSO_4_ 0.001 gL^−1^, MES 7.8 gL^−1^, 0.5% (v/v) glycerol, and was supplemented with trace elements of 0.5% (v/v) Z-Salts (10 mL Z-Salts consisted of ZnSO_4_·7H_2_O 0.001 g, CuSO_4_·5H_2_O 0.001 g, H_3_BO_4_ 0.001 g, (NH_4_)_2_SO_4_ 0.5 g, MnSO_4_·H_2_O 0.001 g, NaMoO_4_·H_2_O 0.001 g) at pH 5.4. Subsequently, positive transformants of *A. tumefacien* and spores of strain D were co-cultured for 48 h at 24 °C on an agar medium containing IMM and AS covered with a sterile cellophane sheet. Finally, the cellophane sheets were transferred and placed on an IMM containing G418. This was followed by their incubation in the dark at 28 °C for 3 d [30]. As shown in Figure S5, strain ∆HY44 possessed fluorescent signals LB-1, LB-2, RB-1 and RB-2 (about 1000 bp), which indicated that the target gene *D8.t287* in strain D was successfully knocked out.

#### 3.6.4. UPLC-MS Analysis of Strains D and ∆HY44

In order to verify function of the gene *D8.t287* in strain D, chemical investigation of the mutant strain ∆HY44 was carried out. The crude extract II of strain ∆HY44 was prepared using the same procedure as that of strain D as described in Section 3.3. Six fractions (Q1–Q6) were obtained using a preparative HPLC column. Before UPLC-MS analysis, 13 samples consisting of eight pure compounds 1–8, a standard mixture (HB) and four fractions (Q3, Q4_,_ H3, H4) were prepared; the final concentration of each sample was 1.0 mgmL^−1^. The conditions for UPLC analysis were as follows: 90–5% solvent A (water containing 0.1% formic acid, linear gradient, 0–15 min) and solvent B (acetonitrile containing 0.1% formic acid) at 0.4 mL·min^−1^ with a reverse-phase column (Phenomenex, 1.7 µm, 150 × 2.1 mm).

## 4. Conclusions

Solid culture of strain D endophytic on the coastal plant *E. chrysantha* Lindl. led to the production of eight aromatic polyketides, which were respectively identified as rubrofusarin B (**1**), alternariol 9-*O*-methyl ether (**2**), fonsecinone D (**3**), asperpyrone A (**4**), asperpyrone D (**5**), fonsecinone B (**6**), fonsecinone A (**7**) and aurasperone A (**8**) by a combination of spectroscopic methods (1D NMR and ESI-MS) and comparison with the literature data. It further certified that the species *A. fumigatus* is one of rich sources of natural products and OSMAC is an effective approach to evoke silent genes to produce more SMs [31]. Bioassay results showed that these aromatic polyketides **1**–**8** exhibit no potent antimicrobial activity against *E. coli*, *S. aureus* and *C. albicans*. Genome and BGC analysis indicated that strain D possesses 22 SM BGCs including seven PKS, 10 NRPS, four terpene and one fungal-RiPP gene clusters. Plausible pathways for biosynthesis of dimeric naphtho-*γ*-pyrones **3**–**8** were firstly proposed in this work and one key gene *D8.t287* encoding KS and AT was characterized by target gene knockout and UPLC-MS analysis.

## Figures and Tables

**Table 1:** Comparison of genome features of strain D with other *A. fumigatus* strains deposited in NCBI database.

Strain	Genome Size (Mb)	G + C%	Gene	tRNA	Hypothetical Protein	Assembly
A1163	29.21	49.5	10,124	180	9929	GCA_000150145.1
Af10	28.76	49.5	-	-	-	GCA_000225625.2
Af293	29.39	49.8	9630	229	9916	GCA_000002655.1
CNM-CM8057	28.32	49.5	8910	-	8910	GCA_012656215.1
D	33.40	51.4	9789	108	4417	GCA_003069565.1
HMR AF 270	29.48	49.2	9730	179	9549	GCA_002234955.1
HMR AF 706	28.23	49.5	9466	180	9284	GCA_002234985.1
ISSFT-021	28.24	49.4	-	-	-	GCA_001643655.1
LMB-35Aa	27.52	50.0	-	-	-	GCA_001715275.2
SGAir0713	29.02	49.6	-	-	-	GCA_005768625.2
Z5	29.36	49.2	-	-	-	GCA_001029325.1

-: not available.

**Table 2:** Antibiotics and Secondary Metabolite Analysis Shell 5.1.2 (antiSMASH) analysis of biosynthetic gene clusters in strain D.

	Region	Type	Gene Cluster Position	Most Similar Gene Cluster	Similarity (%)
Scaffold1	1.1	NRPS	3,405,290–3,461,377	/	/
Scaffold2	2.1	terpene	1,004,114–1,024,548	/	/
Scaffold3	3.1	T3PKS	985,697–1,026,775	/	/
Scaffold4	4.1	terpene	388,980–408,695	terpene	40
Scaffold4	4.2	terpene	2,345,955–2,367,289	/	/
Scaffold5	5.1	T1PKS	1,087,051–1,131,054	polyketide	13
Scaffold5	5.2	T1PKS	2,115,332–2,162,885	polyketide	50
Scaffold6	6.1	T1PKS	252,626–300,167	NRP+polyketide	18
Scaffold7	7.1	fungal-RiPP	280,898–318,613	/	/
Scaffold7	7.2	NRPS	1,415,890–1,477,928	NRP: Cyclic depsipeptide	100
Scaffold8	8.1	T1PKS	160,848–205,614	polyketide	100
Scaffold8	8.2	T1PKS	918,158–961,487	polyketide	100
Scaffold8	8.3	NRPS	1,667,861–1,723,346	/	/
Scaffold9	9.1	NRPS+indole	74,519–134,677	/	/
Scaffold9	9.2	terpene	1,083,778–1,105,346	/	/
Scaffold9	9.3	NRPS-like	1,628,313–16,71,303	/	/
Scaffold11	11.1	NRPS	383,909–428,735	NRP+polyketide	45
Scaffold12	12.1	NRPS-like	821,562–865,410	/	/
Scaffold17	17.1	NRPS-like	305,087–348,950	/	/
Scaffold19	19.1	NRPS	260,997–319,962	NRP	100
Scaffold21	21.1	NRPS-like	36,305–79,148	/	/
Scaffold21	21.2	T1PKS	174,713–214,266	polyketide	62

**Table 3:** BLAST results of polyketide synthase (PKS) biosynthetic genes in strain D by Diamond.

Gene	Gene Position	Hit	Similarity (%)
*D1.t382*	1,271,230–1,272,630	polyketide synthase PksC [*Alternaria alternata*] OAG15814.1	100
*D5.t345*	1105803–1,112,689	polyketide synthase PksJ [*A. alternata*] OAG22978.1	84.5
*D5.t661*	2135332–2,143,251	polyketide synthase PksH [*A. alternata*] OAG13655.1	97.6
*D6.t72*	272,318–280,167	polyketide synthase PksG [*A. alternata*]	95.7
*D6.t701*	2,594,273–2,602,296	putative polyketide synthase [*Fusarium aywerte*]	56.8
*D8.t287*	938,512–942,754	ketoacyl-synt-domain-containing protein [*A. alternata*] OAG24819.1	85.3
*D11.t72*	283,919–289,534	polyketide synthase PksD [*A. alternata*] OAG18885.1	90.6
*D11.t100*	403,909–416,371	polyketide synthase PksB [*A. alternata*]	98.3
*D21.t51*	193,704–202,533	polyketide synthase PksF [*A. alternata*] OAG16734.1	99.4

## Supplementary Materials

The following are available online at https://www.mdpi.com/1660-3397/18/6/324/s1. Isolation procedure of aromatic polyketides **1**–**8,** screening resistance markers, colony morphology of positive transformants, PCR verification of target gene, PCR identification result of ∆HY44, conditions for PCR reaction, and ^1^H NMR and ESI-MS spectra of compounds **1**–**8**.
