# Supplementary material for: Aromatic Polyketides from a Symbiotic Strain Aspergillus fumigatus D and Characterization of Their Biosynthetic Gene D8.t287
Source: Mar Drugs. 2020 Jun 20;18(6):324. doi: 10.3390/md18060324 (PMC7344599; doi:10.3390/md18060324)
Supplement: Supplementary file 1 [file marinedrugs-18-00324-s001.pdf]

Supplementary Materials for

Aromatic Polyketides from a Symbiotic Strain *Aspergillus fumigatus* D and Characterization of Their Biosynthetic Gene *D8.t287*

Yi Hua [1], Rui Pan [1], Xuelian Bai [2], Bin Wei [1], Jianwei Chen [1], Hong Wang [1,*] and Huawei Zhang [1,*]

Figure S1. Isolation procedure of compounds **1-8** 1

Figure S2. Screening resistance marker of strain D 1

Figure S3. PCR verification of target genes LB and RB in constructed vector 1

Figure S4. Colony morphology of positive transformant and PCR verification 1

Figure S5. PCR identification results of mutant strain ΔHY44 2

Figure S6. ^{1}H NMR Spectrum of compound **1** in $CDCl_3$ 3

Figure S7. ^{1}H NMR Spectrum of compound **2** in DMSO-d_6 4

Figure S8. ^{1}H NMR Spectrum of compound **3** in $CDCl_3$ 5

Figure S9. ^{1}H NMR Spectrum of compound **4** in $CDCl_3$ 6

Figure S10. ^{1}H NMR Spectrum of compound **5** in $CDCl_3$ 7

Figure S11. ^{1}H NMR Spectrum of compound **6** in $CDCl_3$ 8

Figure S12. ^{1}H NMR Spectrum of compound **7** in $CDCl_3$ 9

Figure S13. ^{1}H NMR Spectrum of compound **8** in $CDCl_3$ 11

Table S1. Primers and its sequences of PCR 2

Table S2. Conditions of PCR reaction 2

Table S3. Comparison of ^{1}H NMR spectral data of compound **1** 3

Table S4. Comparison of ^{1}H NMR spectral data of compound **2** 4

Table S5. Comparison of ^{1}H NMR spectral data of compound **3** 5

Table S6. Comparison of ^{1}H NMR spectral data of compound **4** 6

Table S7. Comparison of ^{1}H NMR spectral data of compound **5** 7

Table S8. Comparison of ^{1}H NMR spectral data of compound **6** 8

Table S9. Comparison of ^{1}H NMR spectral data of compound **7** 10

Table S10. Comparison of ^{1}H NMR spectral data of compound **8** 11

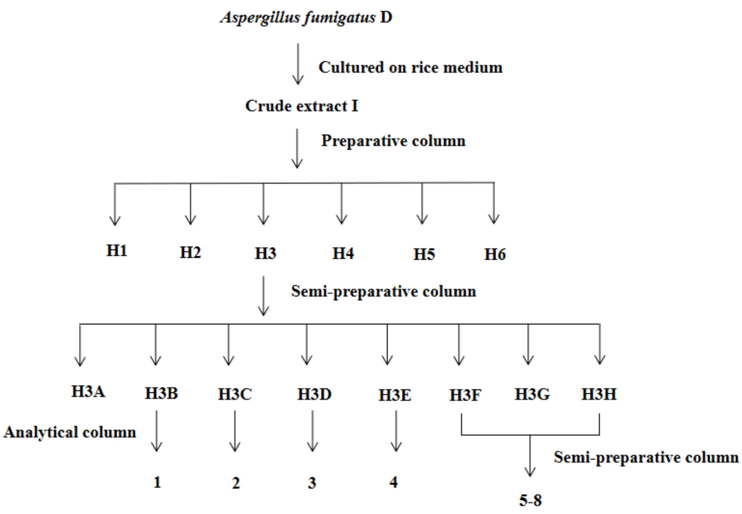

Figure S1. Isolation procedure for aromatic polyketides **1-8**

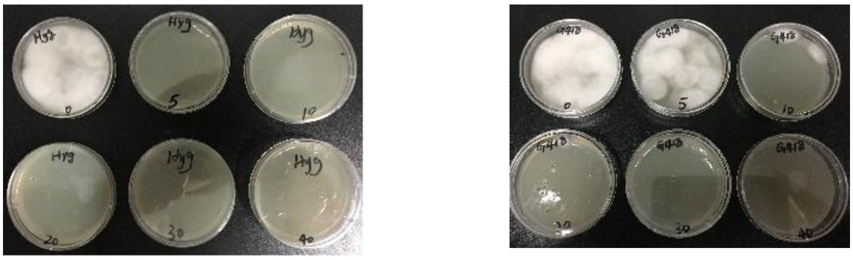

Hyg G418

Figure S2. Screening resistance marker of strain D

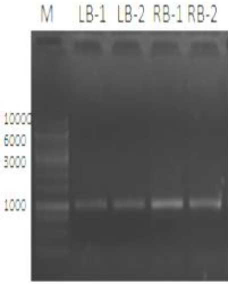

Figure S3. PCR verification of target genes LB and RB in constructed vector

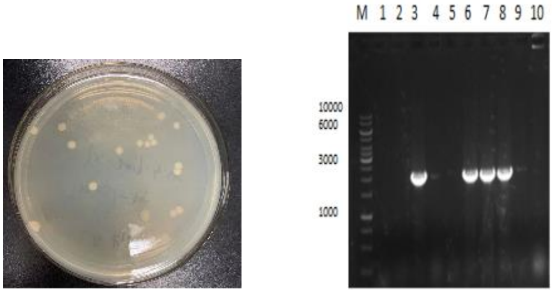

a b

Figure 4. Colony morphology of positive transformant (a) and PCR verification (b, four positive transformants of *E. coli* DH5α).

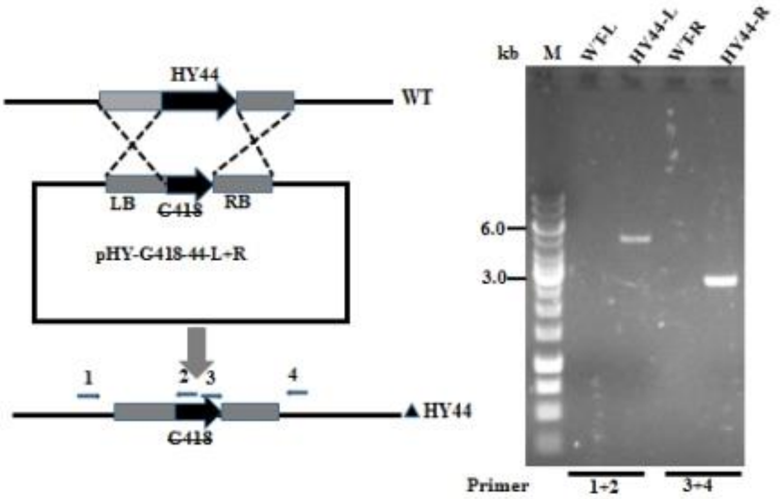

Figure S5. PCR identification of mutant strain ΔHY44

(M: Marker; WT: wild strain; ΔHY44: mutant strain)

Table S1. Conditions for PCR reaction

Reagent	V (μL)
5×PCR Buffer	10
dNTP Mixture	4
P1/ P2 primers	1
Template	1
Primer STAR HS DNA polymerase	1
ddH_2O	33
Total	50

Table S2. Primers and their sequences for PCR verification

Primers	Sequences
HY44-L-1	TTGAACATCGCGCAGTAATGCC
HY44-L-2	GAATTAAATGGTACCGAGCTCGAATTAGACACATTTGGGCAAAGTGCG
HY44-R-1	GTTTAGAGGTAATCCTCCCGGGGACCTCGCTGTTGTGTGCTTTTG
HY44-R-2	CGATCCTAGAACTAGTGGATCCCGTATACTATCGCGAAGAGAAGC
P-check-F	GGCAGGATATATTGTGGTGTAAAC
P-check-R	CTCTCAGTGCGGATCAGTC
P1	GGCAGGATATATTGTGGTGTAAAC
P2	GCCTGCGTTCTATTACTAGCACG
P3	GCATTCGCCATTCAGGCTGC
P4	CGTGACCGATAGCTTCAACACC

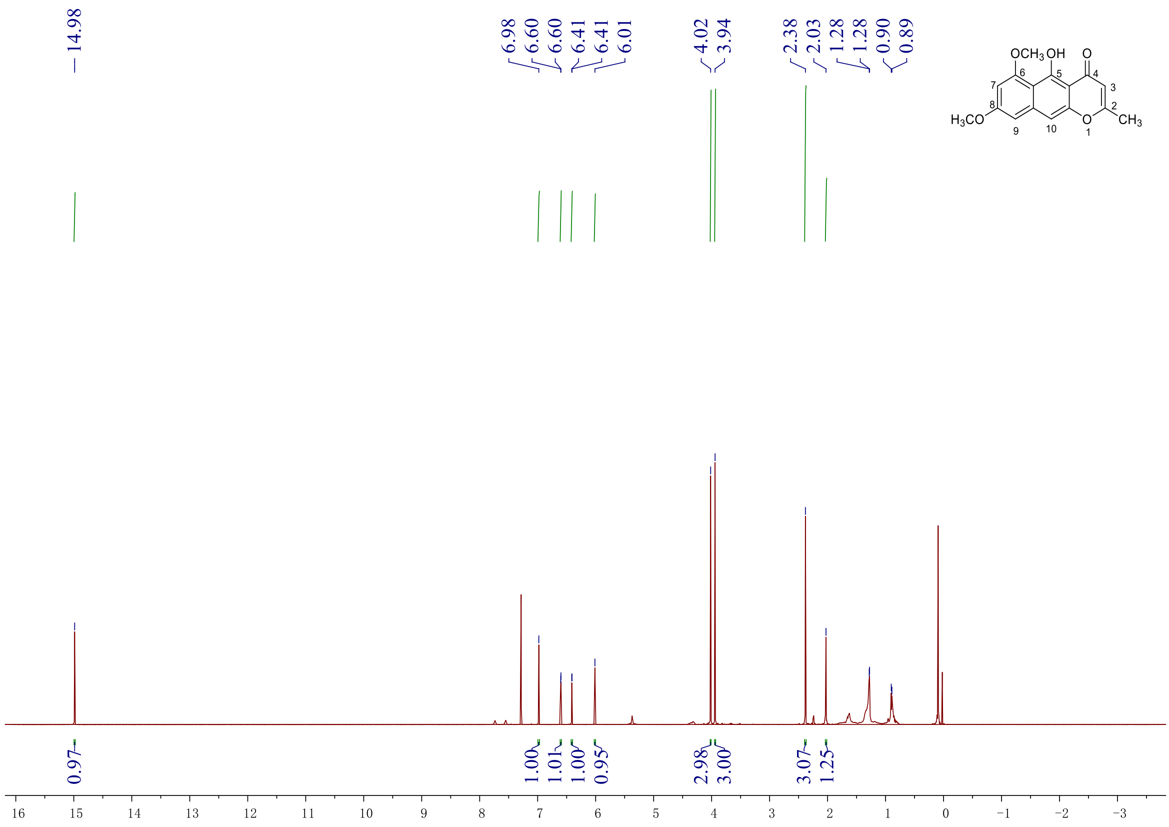

Figure S6. ^{1}H NMR spectrum of compound **1** in $CDCl_3$

Table S3. Comparison of ^{1}H NMR spectral data of compound **1** with reference (600 MHz, $CDCl_3$)

Position	δ_H	δ_H in reference [9]
2	/	/
2-CH_3	2.38 (3H, *s*)	2.37 (3H, *s*)
3	6.01 (1H, *s*)	6.00 (1H, *s*)
4	/	/
5	14.98 (1H, *s*, 5-OH)	14.96 (1H, *s*, 5-OH)
6	/	/
6-OCH_3	4.02 (3H, *s*)	4.00 (3H, *s*)
7	6.41 (1H, *d*, *J* = 2.35)	6.40 (1H, *d*, *J* = 2.20)
8	/	/
8-OCH_3	3.94 (3H, *s*)	3.93 (3H, *s*)
9	6.60 (1H, *d*, *J* = 2.35)	6.59 (1H, *d*, *J* = 2.20)
10	6.98 (1H, *s*)	6.97 (1H, *s*)

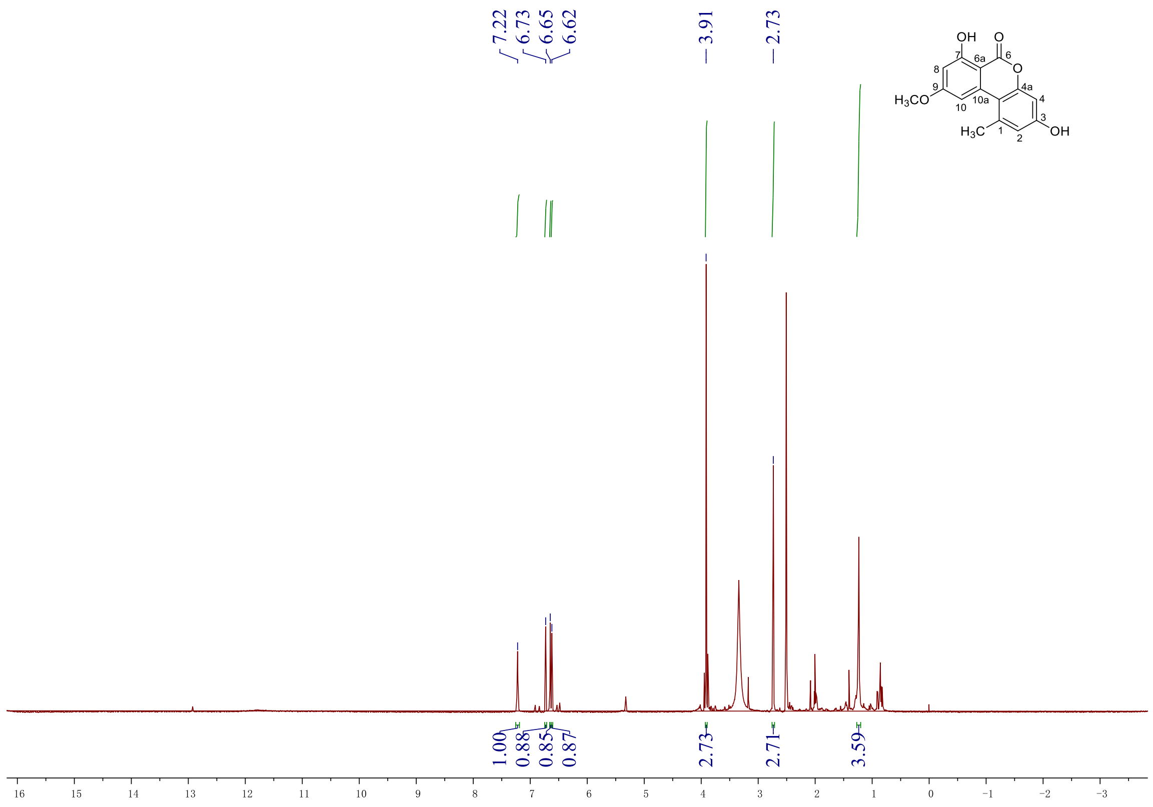

Figure S7. ¹H NMR spectrum of compound **2** in DMSO

Table S4. Comparison of ¹H NMR spectral data of compound **2** with reference (DMSO)

Position	δ_H (*J* in Hz, 600 MHz)	δ_H in reference [10] (*J* in Hz, 500 MHz)
1	/	/
2	6.73 (1H, *d*, *J* = 2.30)	6.73 (1H, *d*, *J* = 2.30)
3	/	/
4	6.65 (1H, *d*, *J* = 2.30)	6.65 (1H, *d*, *J* = 2.30)
4a	/	/
6a	/	/
7	/	/
8	6.62 (1H, *d*, *J* = 1.90)	6.63 (1H, *d*, *J* = 1.90)
9	/	/
10	7.22 (1H, *d*, *J* = 1.90)	7.23 (1H, *d*, *J* = 1.90)
10a	/	/
10b	/	/
11	2.73 (3H, *s*)	2.74 (3H, *s*)
12	3.91 (3H, *s*)	3.91 (3H, *s*)

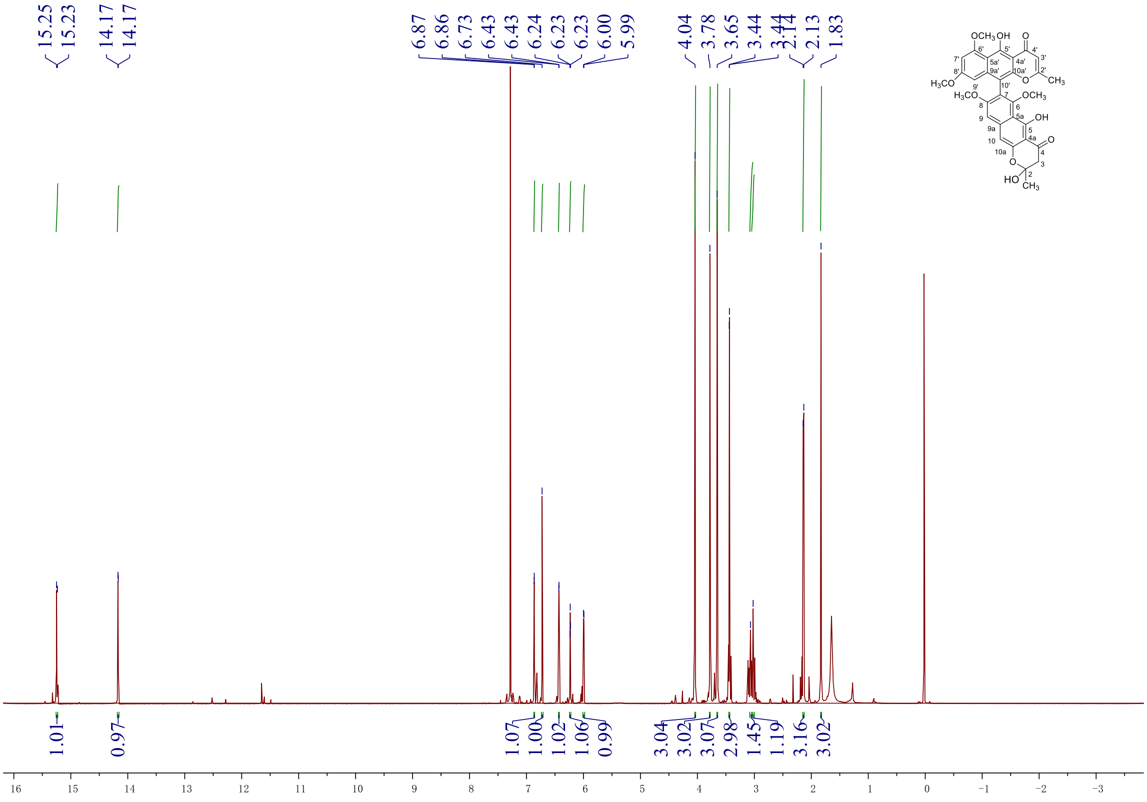

Figure S8. ^{1}H NMR spectrum of compound **3** in $CDCl_3$

Table S5. Comparison of ^{1}H NMR spectral data of compound **3** with reference ($CDCl_3$)

Position	δ_H (*J* in Hz, 600 MHz)	δ_H in reference [11, 12] (*J* in Hz, 60 MHz)
2-CH_3	1.83 (3H, *s*)	1.78 (3H, *s*)
2′-CH_3	2.13 (3H, *s*)	2.11 (3H, *s*)
3	3.02/3.07 (1H, *s*)	3.01 (1H, *s*)
3′	6.00 (1H, *d*, *J* = 4.08)	5.98 (1H, *d*)
5-OH	14.17 (1H, *s*)	14.11 (1H, *s*)
5′-OH	15.24 (1H, *s*)	15.10 (1H, *s*)
6-OMe	3.44 (3H, *s*)	3.42 (3H, *s*)
6′-OMe	4.04 (3H, *s*)	4.00 (3H, *s*)
7′	6.43 (1H, *d*, *J* = 2.09)	6.42 (1H, *d*, *J* = 2.50)
8-OMe	3.78 (3H, *s*)	3.74 (3H, *s*)
8′-OMe	3.65 (3H, *s*)	3.63 (3H, *s*)
9	6.86 (1H, *d*, *J* = 1.41)	6.84 (1H, *d*)
9′	6.23 (1H, *d*, *J* = 2.49)	6.23 (1H, *d*, *J* = 2.50)
10	6.73 (1H, *d*, *J* = 1.41)	6.70 (1H, *d*)

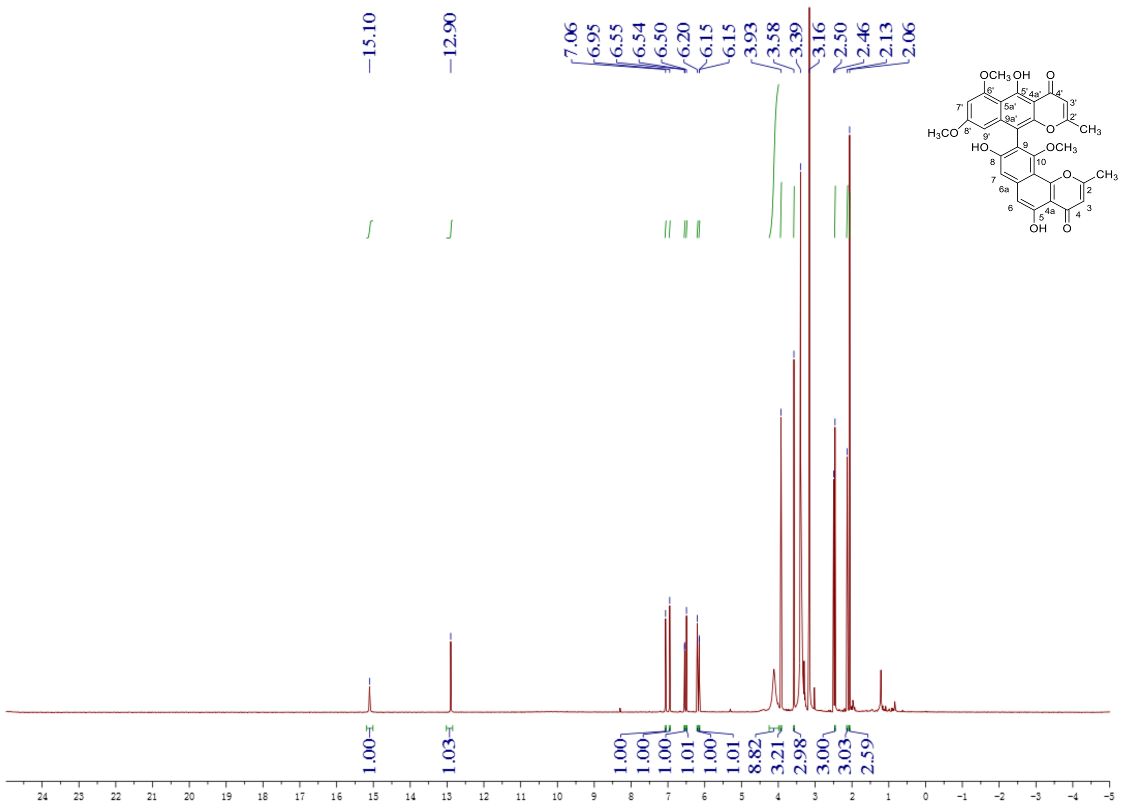

Figure S9. ^{1}H NMR spectrum of compound **4** in $CDCl_3$

Table S6. Comparison of ^{1}H NMR spectral data of compound **4** with reference ($CDCl_3$)

Position	δ_H (*J* in Hz, 600 MHz)	δ_H in reference [13] (*J* in Hz, 500MHz)
2-CH_3	2.48 (3H, *s*)	2.46 (3H, *s*)
2′-CH_3	2.10 (3H, *s*)	2.13 (3H, *s*)
3	6.50 (1H, *s*)	6.50 (1H, *s*)
3′	6.20 (1H, *s*)	6.19 (1H, *s*)
5-OH	12.90 (1H, *s*)	12.90 (1H, *s*)
5′-OH	15.10 (1H, *s*)	15.10 (1H, *s*)
6	6.95 (1H, *s*)	6.96 (1H, *s*)
7	7.06 (1H, *s*)	7.07 (1H, *s*)
7′	6.55 (1H, *d*, *J* = 2.28)	6.55 (1H, *d*, *J* = 2.30)
6′-OMe	3.93 (3H, *s*)	3.93 (3H, *s*)
8′-OMe	3.58 (3H, *s*)	3.58 (3H, *s*)
9′	6.14 (1H, *d*, *J* = 2.29)	6.14 (1H, *d*, *J* = 2.30)
10-OMe	3.39 (3H, *s*)	3.39 (3H, *s*)

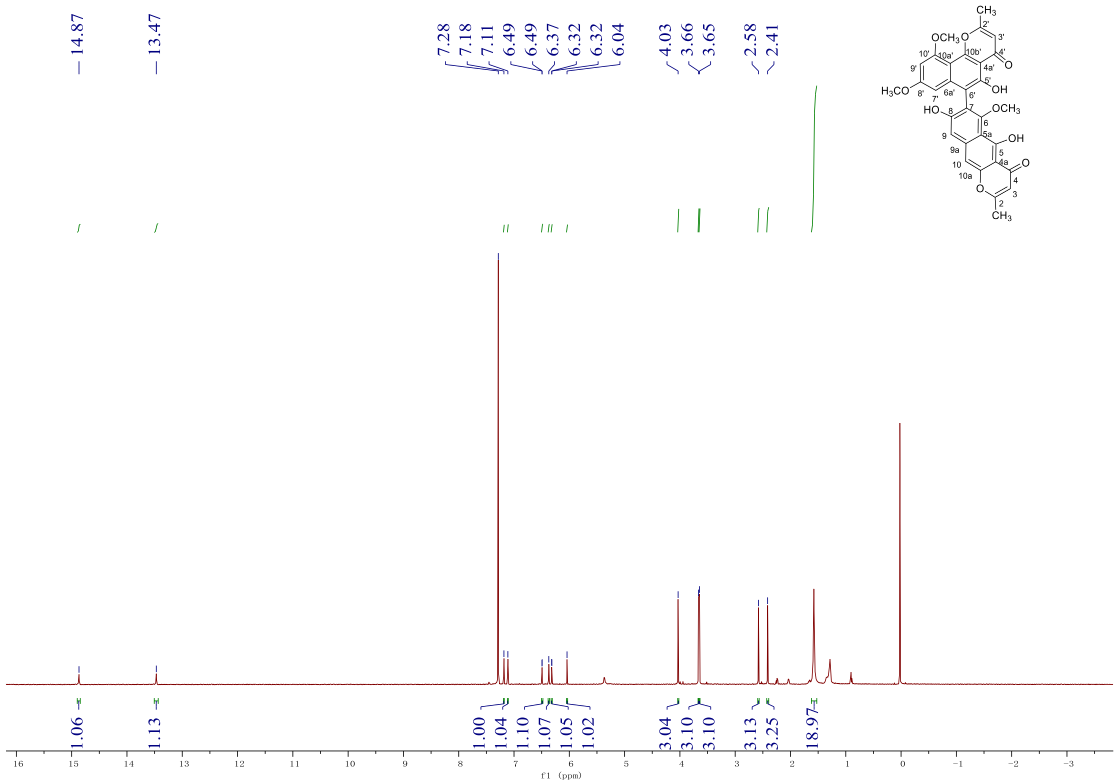

Figure S10. ^1H NMR spectrum of compound **5** in $CDCl_3$

Table S7. Comparison of ^1H NMR spectral data of compound **5** with reference ($CDCl_3$)

Position	δ_H (*J* in Hz, 600 MHz)	δ_H in reference [14] (*J* in Hz, 500 MHz)
2-CH_3	2.41 (3H, *s*)	2.37 (3H, *s*)
2′-CH_3	2.58 (3H, *s*)	2.54 (3H, s)
3	6.04 (1H, *s*)	6.00 (1H, *s*)
3′	6.37 (1H, *s*)	6.33 (1H, *s*)
5-OH	14.87 (1H, *s*)	14.80 (1H, *s*)
5′-OH	13.47 (1H, *s*)	13.40 (1H, *s*)
6-OCH_3	3.66 (3H, *s*)	3.62 (3H, *s*)
7′	6.32 (1H, *d*, *J* = 2.21)	6.27 (1H, *d*, *J* = 2.20)
8′-OMe	3.65 (3H, *s*)	3.60 (3H, *s*)
9	7.18 (1H, *s*)	7.14 (1H, *s*)
9′	6.49 (1H, *d*, *J* = 2.21)	6.45 (1H, *d*, *J* = 2.20)
10	7.11 (1H, *s*)	7.07 (1H, *s*)
10′-OMe	4.03 (3H, *s*)	3.99 (3H, *s*)

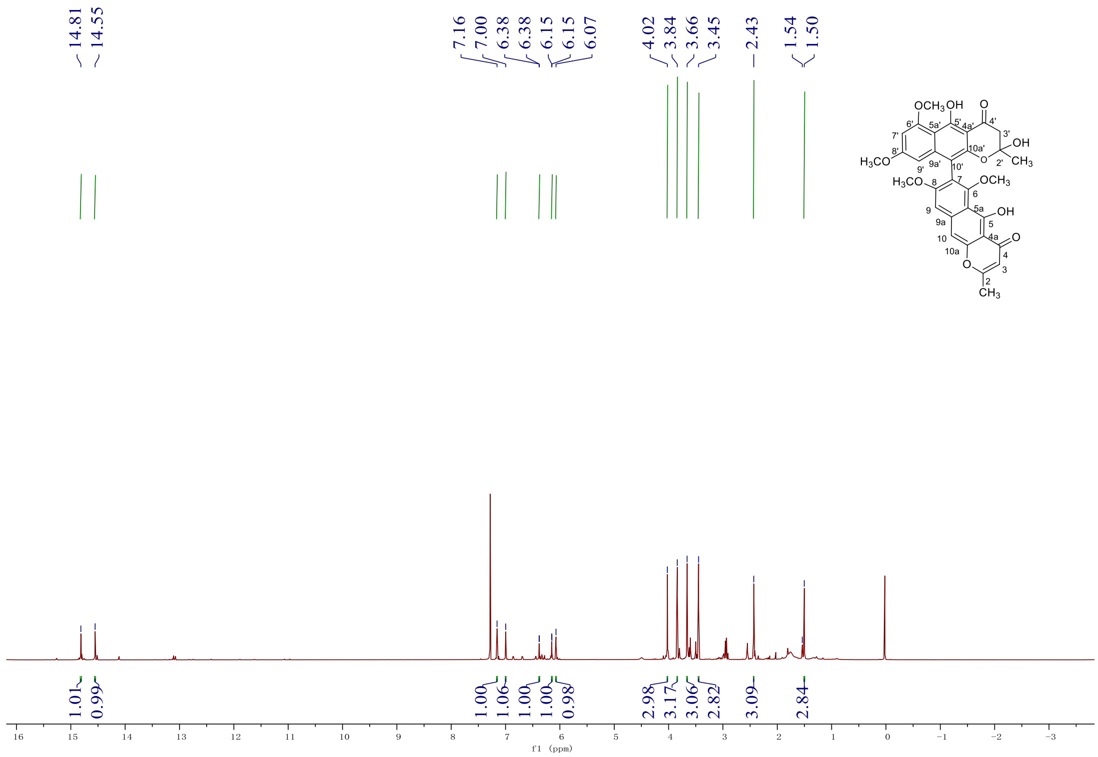

Figure S11. ^{1}H NMR spectrum of compound **6** in $CDCl_3$

Table S8. Comparison of ^{1}H NMR spectral data of compound **6** with reference ($CDCl_3$)

Position	δ_H (*J* in Hz, 600 MHz)	δ_H in reference [11] (*J* in Hz, 60 MHz)
2-CH_3	2.43 (3H, *s*)	2.39 (3H, *s*)
2′-CH_3	1.50 (3H, *s*)	1.49 (3H, *s*)
3	6.07 (1H, *s*)	6.01 (1H, *s*)
3′	3.50 (1H, *s*)	2.91(1H, *s*)
5-OH	14.81 (1H, *s*)	14.70 (1H, *s*)
5′-OH	14.55 (1H, *s*)	14.40 (1H, *s*)
6-OMe	3.45 (3H, *s*)	3.43 (3H, *s*)
6′-OMe	4.02 (3H, *s*)	3.98 (3H, *s*)
7′	6.38 (1H, *d*, *J* = 2.21)	6.35 (1H, *d*, *J* = 2.50)
8-OMe	3.84 (3H, *s*)	3.80 (3H, *s*)
8′-OMe	3.66 (3H, *s*)	3.62 (3H, *s*)
9	7.00 (1H, *s*)	6.95 (1H, *s*)
9′	6.15 (1H, *d*, *J* = 2.21)	6.13 (1H, *d*, *J* = 2.50)
10	7.15 (1H, *s*)	7.10 (1H, s)

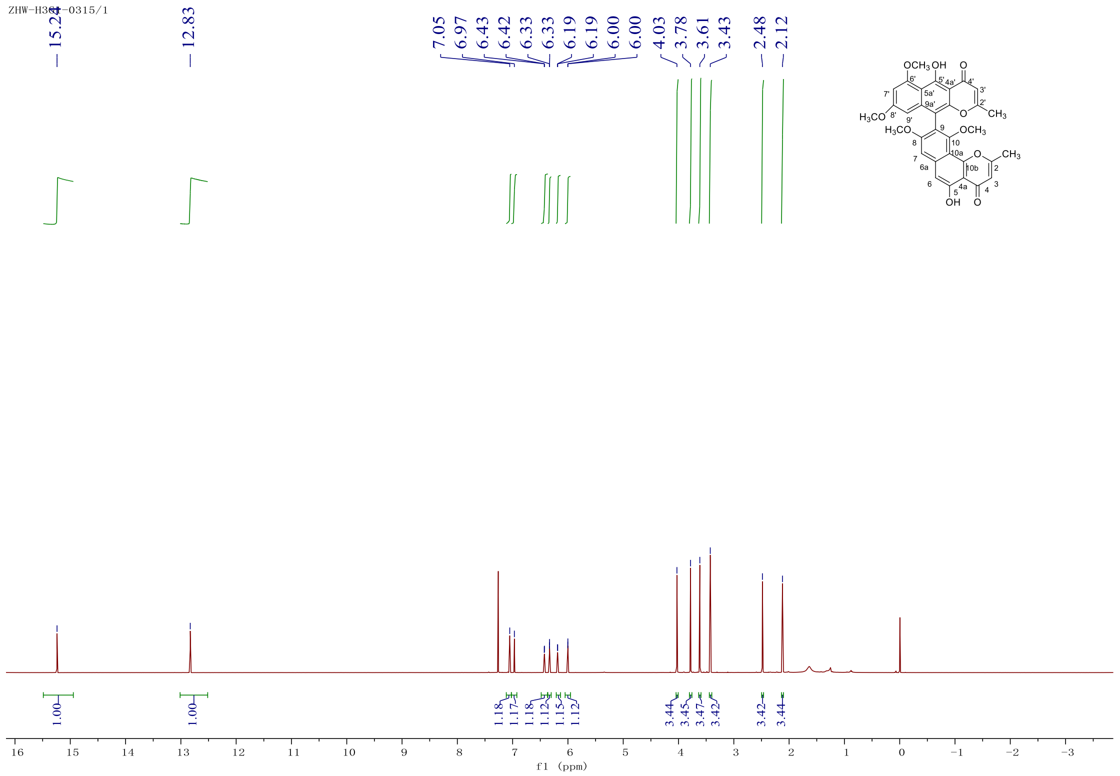

Figure S12. ^{1}H NMR spectrum of compound **7** in $CDCl_3$

Table S9. Comparison of ^{1}H NMR spectral data of compound **7** with reference ($CDCl_3$)

Position	δ_H (*J* in Hz, 600 MHz)	δ_H in reference [15] (*J* in Hz, 400 MHz)
2	/	/
2-CH_3	2.48 (3H, *s*)	2.49 (3H, *s*)
2′	/	/
2′-CH_3	2.12 (3H, *s*)	2.13 (3H, *s*)
3	6.33 (1H, *s*)	6.34 (1H, *s*)
3′	6.00 (1H, *s*)	6.01 (1H, *s*)
4	/	/
4′	/	/
4a	/	/
4a′	/	/
5-OH	12.83 (1H, *s*)	12.85 (1H, *s*)
5′-OH	15.24 (1H, *s*)	15.27 (1H, *s*)
5a′	/	/
6	7.05 (1H, *s*)	7.06 (1H, *s*)
6′	/	/
6′-OMe	4.03 (3H, *s*)	4.04 (3H, *s*)
6a	/	/
7	6.97 (1H, *s*)	6.97 (1H, *s*)
7′	6.43 (1H, *d*, *J* = 2.20)	6.43 (1H, *d*, *J* = 2.20)
8	/	/
8-OMe	3.78 (3H, *s*)	3.79 (3H, *s*)
8′	/	/
8′-OMe	3.61 (3H, *s*)	3.63 (3H, *s*)
9	/	/
9′	6.19 (1H, *d*, *J* = 2.20)	6.19 (1H, *d*, *J* = 2.20)
9a′	/	/
10	/	/
10-OMe	3.43 (3H, *s*)	3.44 (3H, *s*)
10a	/	/
10b	/	/
10′	/	/
10a′	/	/

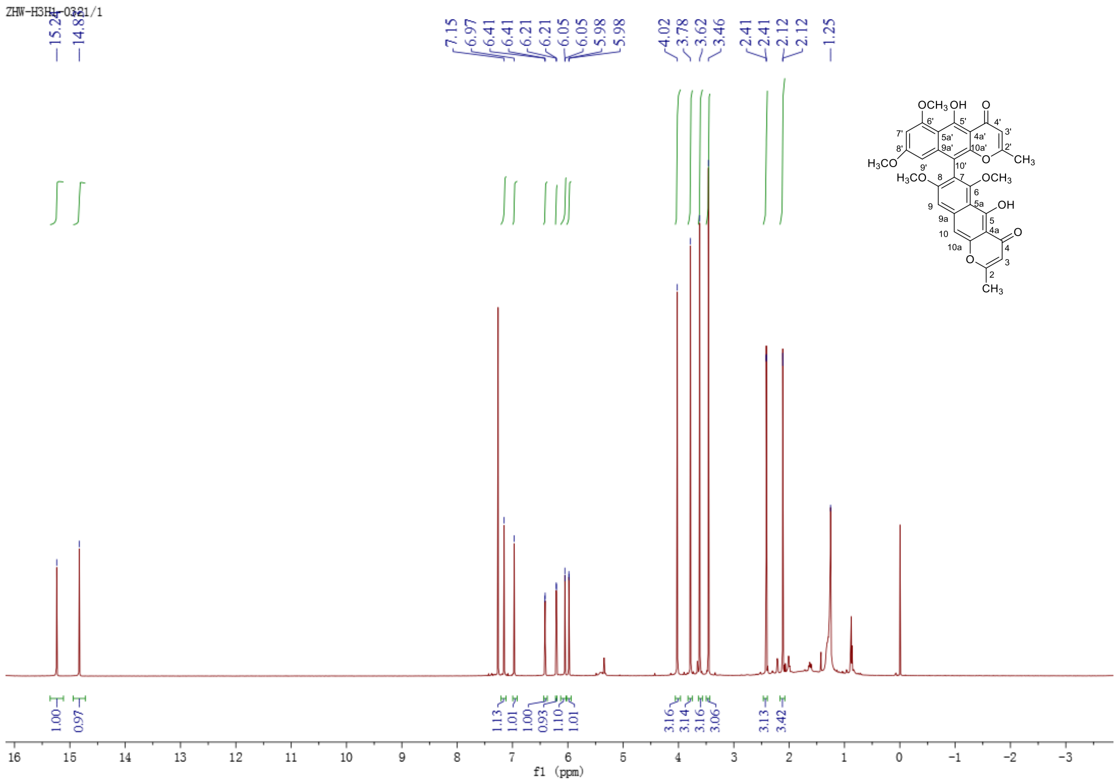

Figure S13. ^{1}H NMR spectrum of compound **8** in $CDCl_3$

Table S10. Comparison of ^{1}H NMR spectral data of compound **8** with reference ($CDCl_3$)

Position	δ_H (J in Hz, 600 MHz)	δ_H in reference [16] (J in Hz, 400 MHz)
2-CH_3	2.41 (3H, *s*)	2.42 (3H, *s*)
2′-CH_3	2.12 (3H, *s*)	2.12 (3H, *s*)
3	6.05 (1H, *s*)	6.06 (1H, *s*)
3′	5.98 (1H, *s*)	5.98 (1H, *s*)
5-OH	14.83 (1H, *s*)	14.84 (1H, *s*)
5′-OH	15.24 (1H, *s*)	15.26 (1H, *s*)
6-OMe	3.46 (1H, *s*)	3.46 (1H, *s*)
6′-OMe	4.02 (3H, *s*)	4.03 (3H, *s*)
7′	6.41 (1H, *d*, J = 2.27)	6.41 (1H, *d*, J = 2.20)
8-OMe	3.78 (3H, *s*)	3.79 (3H, *s*)
8′-OMe	3.62 (3H, *s*)	3.62 (3H, *s*)
9	6.97 (1H, *s*)	6.97 (1H, *s*)
9′	6.20 (1H, *d*, J = 2.27)	6.20 (1H, *d*, J = 2.20)
10	7.15 (1H, *s*)	7.16 (1H, *s*)
